# The effect of wet cupping therapy on the clinical symptoms of adult-onset asthma: a randomized clinical trial

**DOI:** 10.55730/1300-0144.5855

**Published:** 2024-02-17

**Authors:** Abbas JOUSHAN, Hamid Reza HATAMI, Khosrow AGIN, Hoorieh MOHAMMADI KENARI, Sajjad SADEGHI, Rasool CHOOPANI

**Affiliations:** 1 Department of Traditional Medicine, Faculty of Clinical Medicine, Technofest Institute of Technology University, Erquelines, Belgium; 2 Department of Traditional Medicine, School of Traditional Medicine, Shahid Beheshti University of Medical Sciences, Tehran, Iran; 3 Department of Allergy and Clinical Immunology, Faculty of Medicine, Shahid Sadoughi University of Medical Sciences, Yazd, Iran; 4 Department of Adult Pulmonary Diseases, Loghman Hakim Hospital, Shahid Beheshti University of Medical Sciences, Tehran, Iran; 5 Institute for Studies in Medical History, Persian and Complementary Medicine, Iran University of Medical Sciences, Tehran, Iran

**Keywords:** Wet cupping therapy, asthma, bronchial asthma, shortness of breath, Persian medicine

## Abstract

**Background/aim:**

Asthma is an inflammatory disease of the lungs. Cupping therapy is a traditional method used in Persian medicine for treating various ailments. This study aimed to evaluate the anti-asthmatic effects of wet cupping therapy (WCT) in patients with mild to moderate asthma.

**Materials and methods:**

This is a randomized clinical trial conducted on 103 asthma patients who were referred to Loghman Hakim Hospital, Tehran, Iran. The diagnosis of the disease was confirmed by a pulmonologist based on the patient’s history and clinical examinations. The patients who were treated with common asthma medications were assigned to intervention and control groups. The intervention group underwent one session of WCT in the region between two shoulders on one of the 17th, 19th, and 21st days of the lunar month. The clinical signs of all patients were gathered based on the asthma control test questionnaire before the intervention and in the first, second, fourth, sixth, and eighth weeks after the intervention. The scores of the five questionnaire items and the mean total treatment score (MTTS) were compared between the two groups. Additionally, the satisfaction scores of the participants in the two groups were compared.

**Results:**

Of 103 patients, 82 patients completed the study. The mean total treatment score (MTTS) was not significantly different between the control and intervention groups at the beginning of the study (p = 0.06). In the intervention group, the MTTS was 11.44 before WCT, while it was significantly increased (24.24) eighth week after the intervention (p < 0.001). However, the MTTS in the intervention group was significantly higher than the control group in the first week (p <0.001). In addition, at the end of the trial, the subjects’ satisfaction scores in the WCT and control groups were 7.48 and 4.53, respectively (p < 0.001).

**Conclusion:**

Wet cupping therapy can be an efficient therapeutic method to ameliorate respiratory complications of asthma patients.

## 1. Introduction

Asthma is an inflammatory disease of the airways, accompanied by reversible bronchoconstriction and symptoms such as coughing and shortness of breath [1]. It is estimated that the prevalence of asthma in different countries’ populations can be as high as 18%. By 2025, asthma, a common chronic disease, is expected to affect approximately 400 million people worldwide [2,3]. Current treatments for asthma, including class *β*2 agonist drugs, anticholinergics, corticosteroids and xanthine drugs, fail in some patients due to complicated pathophysiology of asthma or lack of medication adherence. The high economic and social burden of asthma, as well as its high prevalence and adverse effects, necessitates seeking supplementary treatments for the disease [4,5]. There is a growing global trend toward Traditional and Complementary Medicine for respiratory disorders, including asthma. Studies have been published on the beneficial effects of herbal medicines, natural and food-based products, and manual treatments like acupuncture and cupping therapy [5–7].

Cupping therapy is a well-known manual method used in Persian medicine for treating a variety of diseases. It is generally divided into two types: wet cupping and dry cupping [8]. Dry cupping involves applying negative pressure sucking on the skin without any bloodletting [9]. In contrast, wet cupping therapy (WCT) involves bloodletting, similar to venesection (Fasd) and leech therapy. As described in Persian medicine, WCT involves superficial scarification of the congested skin followed by brief suction to facilitate bloodletting [10]. According to previous reports, WCT is an effective therapeutic technique for treating various conditions, including neck pain, low back pain, carpal tunnel syndrome, herpes zoster, and knee osteoarthritis [11–15]. This method is also applicable for treating acute and chronic inflammation, as well as diseases of the immune system. WCT has been shown to alleviate oxidative stress and modulate the release of inflammatory cytokines, resulting in the regulation of the immune system [16]. Moreover, an animal study showed that WCT has antiasthmatic effects by reducing eosinophil trafficking and modulating Th2 inflammatory cytokines [17].

This therapeutic method has been increasingly accepted by different cultures and people around the world. Several advantages of cupping therapy, such as its extensive and easy application, good efficacy, low cost, and safety, have encouraged many practitioners to introduce this technique into their therapeutic practices [18]. Considering the benefits of WCT for respiratory disorders reported in previous studies [17,19], this randomized clinical trial was designed to investigate the effect of WCT on the clinical symptoms of adult-onset asthma in patients with mild to moderate asthma, aged 18–60 years.

## 2. Materials and methods

### 2.1. Study design and participants

This randomized clinical trial was performed on patients with mild to moderate asthma, aged 18–60 years, who were referred to the Respiratory Diseases Clinic of Loghman Hospital, Tehran, Iran between August 1 and November 6, 2020. The study was approved by the local ethics committee of Shahid Beheshti University of Medical Sciences in Tehran, Iran in 2020 (ethical code: IR.SBMU.RETECH.REC.1399.351) and adhered to the Declaration of Helsinki. Additionally, the clinical trial was registered with the Iranian Registry of Clinical Trials (registration code: IRCT20181110041600N1). All participants provided written consent and were informed about the purpose and method of the study. Personal information of the volunteers was kept confidential. In the event of any complications or treatment issues, necessary instructions were provided to the participants.

The inclusion criteria were as follows: patients with mild to moderate asthma who had stable conditions without hospitalization, were between 18 and 60 years old, consented to participate in the study, and had no underlying diseases such as cystic fibrosis, bronchopulmonary disease, dysplasia, heart failure, pulmonary embolism, tracheobronchomalacia, bronchiectasis, sarcoidosis, or diabetes. Additionally, participants were required to be free of medications such as aspirin, beta-blockers, and NSAIDs, to have not received cupping therapy in the previous month, and to have no history of coagulation disorders. The exclusion criteria were as follows: patients who were unable to express the severity of their symptoms, required hospitalization, had a full or empty stomach at the time of therapy, were menstruating, engaged in sexual activity within the last 12 hours before wet cupping, had anemia, immune deficiency, smoked, were breastfeeding, or were pregnant.

### 2.2. Outcome measurements

Data collection was carried out through interviews, examinations, patient information forms, cupping complications forms, and the standard asthma control test (ACT) questionnaire. The ACT questionnaire consists of 5 questions regarding the frequency of asthma symptoms and any medication used in the previous 4 weeks. The questionnaire encompassed questions related to daily activities, the frequency of shortness of breath, nocturnal asthma symptoms, the necessity for rescue inhaler or nebulizer medication, and an assessment of the patient’s asthma control. The scores on this test are based on a Likert scale ranging from 5 (poor control) to 25 (complete control). Scores for controlled asthma, not well-controlled asthma, and uncontrolled asthma are ≥20, 16–19, and ≤15, respectively [20]. It should be noted that the ACT questionnaire is a valid and widely accepted tool for evaluating asthmatic patients in research studies. In our study, we utilized the Persian version of this questionnaire, which has been approved by the Ministry of Health and Medical Education of Iran [21]. Furthermore, patient satisfaction with the intervention in each group was measured at the end of the trial using the VAS scale (0–10).

### 2.3. Interventions

According to this study’s protocol, both groups were treated with common asthma medications, including fluticasone (25.25 μg, two puffs twice a day) and salbutamol sprays (250 mg/kg) (if needed). The patients in the intervention group were also treated with a wet cupping session on one of the 17th, 19th, or 21th day of lunar month.

**Figure 1:**
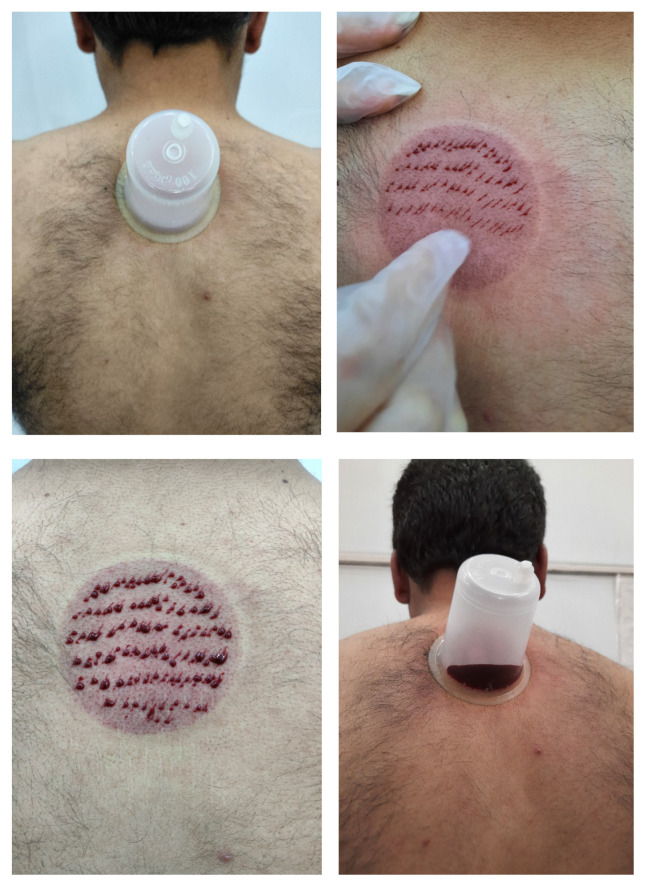
Wet cupping sites and steps.

To perform wet cupping, the patient was seated cross-legged on the bed, and the practitioner applied an antiseptic agent, such as alcohol, to disinfect a specific site on the body where cupping would take place. The cupping site was the patient’s back, specifically the area between the two scapulae, situated between the second and fifth thoracic vertebrae [22]. Next, the cup was placed on the skin, and a vacuum was created in the cup using a suction device. This vacuum, combined with the environmental air pressure, caused the skin to be pulled into the cup, forming a skin dome with a height of 1–1.5 cm. After 3–5 min, the cup was removed, and 10–15 skin-deep scars (0.5 to 1 mm) were made using a surgical razor. The cup was then reapplied to the same spot, and the suction process was continued to gradually draw out the blood. Afterward, the suction device was removed from the cup, and the cycle was repeated 3 times, each lasting 5 min (Figure 1). It is noteworthy that the patient’s position during cupping was in accordance with Iranian customs, with the patient sitting. This seated position facilitated the removal and reapplication of the cup, making the cupping process more convenient for the practitioner.

Finally, postcupping recommendations were given to the patient, which included: avoiding exercise and strenuous physical activity, as well as refraining from eating eggs, fish, dairy products (yogurt, milk, buttermilk), salty foods, and fried foods for up to 24 h after cupping; abstaining from sexual intercourse up to 24 h; and avoiding sleeping immediately after cupping. Notably, this WCT protocol was designed based on Persian Medicine resources and Islamic medical recommendations [8,23].

Patients in both groups were visited and evaluated by a pulmonologist in the first, fourth, and eighth weeks of the study, during which the ACT questionnaire was completed. Moreover, all patients were followed up by phone call at the end of the first, second, and sixth weeks, during which the ACT questionnaire was again completed for each patient to determine the status of asthma control. The groups were then compared. A researcher-deigned questionnaire regarding the complications of wet cupping was also provided to the patients in the intervention group to record any complications.

### 2.4. Sample size and statistical analysis

Assuming the detection of at least a 5-point difference in the ACT measurement scores between the control and experimental groups (2 points in standard drug treatment group and 7 points in the cupping therapy group), with an effect size of 0.25, a common variance of 5, a test power of 80%, and significance level of 0.05, the sample size was estimated as 30 subjects for each group. The formula used for calculating the sample size is as follows:


$$\text{n}=\frac{2.{\left({\text{Z}}_{\alpha /2}+{\text{Z}}_{\beta }\right)}^{2}.\text{Var}}{\text{Effect }{\text{Size}}^{2}}$$

Finally, considering a 20% withdrawal rate, the total calculated sample size was 72 patients (36 patients in each group). Using a random block procedure, 103 patients (51 patients in the control group and 52 subjects in the intervention group) were included in the study, meeting the inclusion criteria and providing informed consent.

After collecting the questionnaire information, data were analyzed using SPSS software version 22 through descriptive and inferential statistics. Descriptive statistics, including frequency, percentage, and mean ± standard deviation, were used to summarize and describe the data. The independent t-test was employed for analyzing quantitative variables. p-values of less than 0.05 were considered statistically significant.

## 3. Results

In this study, 103 subjects were recruited and divided randomly into two groups (52 subjects in the WCT and 51 in the control groups). Among them, 82 patients completed the trial, with 41 in each group. In the intervention group, 25 out of 52 patients (48.1%) were male and 27 (51.9%) were female. In the control group, 12 out of 51 patients (23.5%) were male and 39 (76.5%) were female. The mean age of participants in the intervention group was 44.34 years, while in the control group it was 43.92 years. Comparing the marital status distribution of patients, the intervention group had 46 (88.5%) married and 6 (11.5%) single, while in the control group had 41 (80.4%) married and 10 (19.6%) single. Besides, the two groups did not differ significantly regarding their educational status (p = 0.51). In terms of demographic variables, there were no statistically significant differences between the two groups, except for sex, disease history, and other diseases (p < 0.05; Table 1).

### 3.1. Outcomes

**Figure 2:**
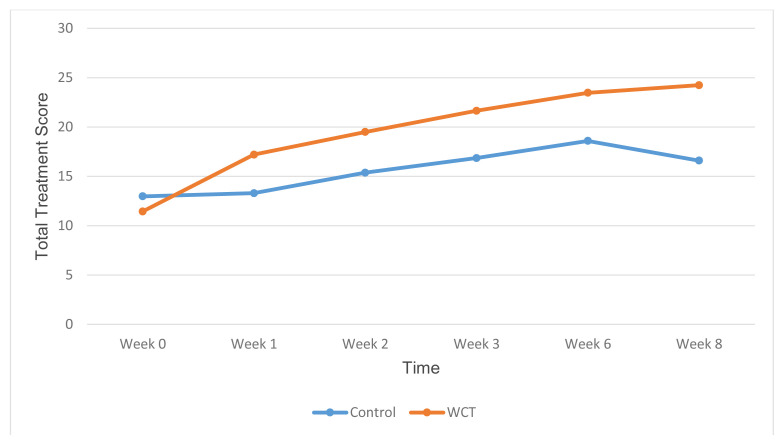
Total treatment score status based on ACT questionnaire.

Patients’ answers to the questions in the ACT questionnaire were recorded and evaluated for zero, first, second, fourth, sixth, and eighth weeks of the study. The mean total treatment score (MTTS) in the intervention group at the eighth week posttreatment (24.2) was significantly higher than the score at the first week (11.44; p < 0.001). The MTTS between the control and intervention groups are shown in Figure 2. The MTTS in all posttreatment visits, except for week 0, was significantly higher in the intervention group than in the control group (p < 0.001). Data from the ACT questionnaire showed that the total treatment score in the wet cupping group increased during the 8 weeks of the study, rising from the out-of-control range (11.44) to complete control of symptoms (24.24).

The results showed that WCT significantly increased the ability of the patients to perform their normal tasks at work, school, or home. Therapeutic efficacy of WCT in alleviating asthma complications gradually appeared from the first to the eighth week posttreatment. Approximately 100% of patients in the WCT group had no asthma-related complication at the end of the eighth week. However, 53.7% of the patients in the control group still experienced some difficulties with asthma symptoms and daily activities during this period. WCT also significantly improved the shortness of breath in these patients. Based on our findings, 97.6% of patients receiving wet cupping had no complaints of shortness of breath by the end of the eighth week, compared to 53.8% who had experienced this issue more than once a day at week zero. Conversely, only 30% of the patients in the control group had no signs of shortness of breath.

Another positive finding was the effect of cupping therapy on improving nocturnal symptoms in patients with asthma. Although 11.5% of patients in the cupping group had high quality sleep at week zero, the sleep quality of all patients treated with cupping method remained unchanged at the end of the eighth. Nonetheless, about 70% of patients in the control group complained of poor sleep quality due to asthma problems. Further evaluations indicated that cupping therapy had a significant effect on reducing the use of asthma medications by patients over the study period. According to the present data, only 3.8% of patients in the intervention group did not need to use inhaled asthma medications at week zero, while more than 50% of them did not use these drugs at the end of the eighth week. Additionally, the need for rescue inhalers (salbutamol) was significantly lower in the WCT group than in the control group (p < 0.001). Specifically, 56.1% of the subjects in the WCT group did not use salbutamol, while this rate was only 19.5% in the control group.

The results of asthma control status showed that patients receiving WCT achieved complete control of asthma in comparison to the control group. None of the patients had complete control of asthma at the beginning of the first week, but at the end of the eighth week, 75.6% of patients in the intervention group and 7.3% in the control group. Detailed information pertaining to the ACT questionnaire is summarized in Table 2. Finally, at the end of the trial, the satisfaction scores for subjects in the WCT and the control groups were 7.48 and 4.53, respectively (p < 0.001).

### 3.2. Treatment safety

Assessment of side effects of cupping therapy revealed no instances of bruising, ecchymosis, blistering, weakness and lethargy, or fainting during the application of cup followed by suction, skin scarification, and bleeding. Only mild scarring at the cupping site was reported in 2 patients. No weakness, lethargy, or fainting was observed after medicinal bleeding in 97.6% of the patients. One case of weakness and lethargy (2.4%) was noted, which improved with supportive measures.

## 4. Discussion

Wet cupping therapy is a traditional method used for treating various diseases [24]. Despite the significance of this therapeutic method and various reports on the effectiveness of cupping [25], the present study was conducted to investigate the effect of wet cupping on the clinical symptoms of adult-onset asthma. Based on the results, WCT effectively controlled the clinical symptoms in patients with mild to moderate asthma. Compared to the control group, patients who underwent wet cupping experienced better symptom control, higher sleep quality, fewer complications, and a reduced need for rescue inhalers.

Numerous studies have examined the effect of cupping on respiratory diseases. For instance, Abd al-Jawad et al. conducted a clinical trial showing that wet cupping, as an adjuvant therapy, cloud improve clinical symptoms of asthma (including daytime symptoms, nocturnal symptoms, need for reliever medication, and the number of exacerbations), as well as patients’ preclinical profiles (including FEV1, FVC %, FEV1/FVC, and FEF25–75%) [19]. Compared to our study, which included a larger sample size and administeres WCT on specific days with a different protocol, Abd al-Jawad’s study used a 3-point wet cupping approach conducted over 3 sessions. In contrast, our study utilized a one-point cupping method, which appears to be more patient-friendly.

Hekmatpou et al. designed a randomized controlled clinical study to compare the effects of wet cupping on arterial O_2_ saturation level in cigarette smokers with venesection. Their results suggested that wet cupping positively affected O2 saturation, leading to improved respiration both during the intervention and posttreatment [26]. Additionally, a study conducted by Ting et al. uncovered that the positive effect of cupping therapy on O_2_ saturation is attributed to its effects on increasing the concentration of oxyhemoglobin (HbO_2_) and reducing the level of deoxyhemoglobin (Hb) in the tissue surrounding the cupping location [27]. Sungchul and colleagues had similar observations regarding tissue oxygenation following cupping therapy [28]. These data are consistent with the findings of the present study, which also noted improvements in the respiration quality of patients. Additionally, a study conducted by Kordafshari et al. examined the effect of cupping on quality of life [29]. Results of the study indicated a positive effect of cupping on improving the quality of life of the subjects, which is in line with the findings of our study. In addition, a randomized clinical trial evaluating the effect of cupping therapy on patients with suboptimal health status and body pain demonstrated the method’s efficacy in enhancing the patients’ quality of life [30].

Chronic inflammatory responses in the lungs and airways are involved in the development and progression of asthma. Therefore, any approach that can block these responses is crucial for alleviating symptoms in asthma patients [31]. Tagil et al. showed that WTC effectively removed oxidative stress-induced products and, as a result, reduced inflammatory complications in a study involving 31 healthy participants [16]. A recently published animal study revealed that WCT could decrease eosinophil counts and interleukin levels in the Balb/c mice model of allergic asthma. Moreover, histological findings revealed that wet cupping has decreased lung tissue inflammation compared to the control group. [17]. Oxidative stress and its related factors and products play a major role in inducing inflammatory processes [32]. Therefore, removing these oxidants and decreasing oxidative stress in the body may ameliorate inflammatory responses and help improve the quality of life for patients with inflammatory diseases such as asthma. Previous data also support that the severity of asthma is associated with decreased antioxidant defenses and increased oxidative mechanisms [33–35]. However, this may be one of the potential mechanisms that can be targeted by the cupping method to tackle the asthma-associated adverse effects. Therefore, further clinical trials are needed to establish the therapeutic potential of this technique in treating asthma or at least in reducing its adverse effects.

In this trial, the timing for performing wet cupping was adjusted according to Persian Medicine resources and Islamic texts. Accordingly, WCT was performed only on the 17th, 19th, and 21st days of the lunar month, which slightly limited participant recruitment. Traditional medicine holds that cupping performed during the initial days of the second half of the lunar month extracts more concentrated substances from the blood. This phenomenon is believed to be influenced by the moon’s gravitational effect on blood circulation [8]. Several studies have demonstrated the potential impact of lunar cycles on the physiology and behavior of both humans and certain animals [36]. Additionally, some studies have indicated that wet cupping therapy performed during the second half of the lunar cycle may be more beneficial [37,38]. However, to draw accurate conclusions, it is necessary to conduct further studies comparing the effects of WCT on asthma on specific lunar days with those on regular days.

In this study, a gender disparity was observed between the two groups, with a higher number of men in the intervention group. This difference can be attributed to the higher prevalence of exclusion criteria for women, such as anemia and menstruation, as well as lower interest among women in the intervention group regarding WCT. While recent research has explored the impact of gender and sex on the pathogenesis and effective management of asthma [3], it is noteworthy that current asthma guidelines do not delineate separate protocols for women and men [39]. Furthermore, in previous studies on WCT, its distinct effects on both men and women were not clearly established [40]. Consequently, no definitive conclusions can be drawn regarding its gender-specific effects.

One of the strengths of the present study was the two-month follow-up with six visits, which provided a more accurate assessment of the gradual changes in WCT compared to the control group. A limitation of the study was the lack of paraclinical tools, such as respiratory function and laboratory tests. Given the positive effects of WCT observed in this study, it is recommended that future research consider incorporating these additional assessments to further evaluate its efficacy.

## 5. Conclusion

Performing a wet cupping session on the 17th, 19th, or 21st day of the lunar month, in conjunction with conventional medications, may improve respiratory function and reduce the need for asthma medications. This therapeutic approach also could increase treatment satisfaction, improve sleep quality, daily activities, and ultimately quality of life in patients with asthma. Therefore, WTC can be considered a promising complementary and therapeutic modality for patients suffering from asthma.

## Figures and Tables

**Table 1:** Comparison of demographic variables between the two groups.

Variable	Control		Wet cupping		p-value
Average age	43.92		44.34		0.84
Sex distribution	Male	Female	Male	Female	0.009
	23.5%	76.5%	48.1%	51.9%	
Body mass index (kg/m^2^)	29.15		28.20		0.38
The year of the disease	3.33		>5.0		0.038
The frequency of other diseases (nonrespiratory)	56.9%		76.9%		0.03

**Table 2:** Comparison of the frequency of ACT questionnaire items between groups at the end of 4th and 8th weeks of the study.

Question number-Subject area	Items	Participant responses at week 4 (%)			Participants responses at week 8 (%)		p-value
		Control	Wet cupping therapy	p-value	Control	Wet cupping therapy	
1-Disturbances of daily activities	Always	4.8	0	0.001	0	0	<0.001
	Often	2.4	0		0	0	
	Sometimes	19	0		9.8	0	
	Rarely	33.3	0.15		43.9	0	
	Never	40.5	0.85		46.3	100	
2- Frequency of shortness of breath	More than once a day	9.5	2.5	<0.001	12.2	0	
	Once a day	16.7	0		4.9	0	
	3–6 times a week	11.9	2.5		7.3	0	
	Once or twice a week	47.6	17.5		46.3	2.4	
	Not at all	14.3	77.5		29.3	97.6	
3- Nocturnal symptoms	4 or more nights a week	7.1	0	0.09	0	0	0.005
	2 or 3 nights a week	4.8	2.5		2.4	0	
	Once a week	31	15		2.4	0	
	Once or twice	0	2.5		22	0	
	Not at all	57.1	80		73.2	100	
4- Using rescue inhaler or nebulizer medication	3 or more times a day	0	0	<0.001	2.4	0	<0.001
	Once or twice a day	88.1	20		78.1	0	
	2 or 3 times a week	7.1	27.5		0	2.4	
	Once a week or less	0	40		0	41.5	
	Not at all	4.8	12.5		19.5	56.1	
5- Asthma control	Not controlled at all	2.4	0	<0.001			<0.001
	Poorly controlled	11.9	0				
	Somewhat controlled	40.5	7.5		22	0	
	Well controlled	40.5	90		70.7	24.4	
	Completely controlled	4.8	2.5		7.3	75.6	

## Abbreviations

WCT: wet cupping therapy
ACT: asthma control test questionnaire
MTTS: mean total treatment score
IRCT: Iranian Registry of Clinical Trials
